# First report of *Corynespora cassiicola* causing leaf spot on *Begonia fimbristipula* in China

**DOI:** 10.1371/journal.pone.0346996

**Published:** 2026-07-30

**Authors:** Xiaobo Zhang, Xiaoyi Zuo, Jianhua Lu, Ya Xiao, Yuanshun Wang, Huan Chen, Yuting Zhang, Xun Lu

**Affiliations:** 1 Kaili University, Kaili, Guizhou Province, People’s Republic of China; 2 Agricultural Science Institute of Xiangxi Tujia and Miao Autonomous Prefecture, Jishou, Hunan Province, People’s Republic of China; 3 Public Resource Centre of XiangXi Tujia and Miao Autonomous Prefecture, Jishou, Hunan Province, People’s Republic of China; University of Duhok, IRAQ

## Abstract

**Background:**

*Begonia fimbristipula*, an economically important medicinal plant in southern China, has recently suffered severe yield losses from leaf spot disease in Guizhou and Hunan Provinces; the causal pathogen remains unknown.

**Methods:**

Field surveys were conducted across multiple locations in Guizhou and Hunan Provinces to record disease symptoms and incidence. The pathogen was isolated from diseased leaves via tissue culture, and identified by combining morphological characteristics and multilocus phylogenetic analysis (ITS, TEF1α, RPB2). Pathogenicity was verified by fulfilling Koch’s postulates through artificial inoculation and re-isolation assays.

**Results:**

Severe leaf spot outbreaks were observed in August 2024, with over 60% disease incidence across 667 hectares and >20% fresh leaf yield loss. The isolated pathogen was identified as Corynespora cassiicola based on gray to dark-gray colonies, pale-brown olivaceous conidia with 0–15 pseudosepta, and ≥98% bootstrap support in phylogenetic clustering with known C. cassiicola strains. Koch's postulates were fulfilled by reproducing typical field symptoms on inoculated B. fimbristipula seedlings and re-isolating the pathogen.

**Conclusions:**

This is the first report of C. cassiicola causing leaf spot on B. fimbristipula in China, expanding the host range of this pathogen. Our findings provide a critical basis for the accurate diagnosis of this newly emerging disease on the medicinal plant.

## Introduction

*Begonia fimbristipula* Hance (Zibeitiankui, a medicinal begonia), a perennial herb belonging to the Begoniaceae family, is widely distributed in southern China and valued for its medicinal properties, including anti-inflammatory and respiratory disease treatment effects [1]. In recent years, the large-scale cultivation of B. fimbristipula has been promoted for its economic value and medicinal properties, including anti-inflammatory and respiratory therapeutic uses, but plant diseases have become a major limiting factor for its production.

In August 2024, a severe leaf spot disease was observed on B. fimbristipula in Huangping County (Guizhou Province) and Guzhang County (Hunan Province), leading to extensive defoliation and yield losses estimated at over 20%. *Corynespora cassiicola* (Berk. & M.A. Curtis) C.T. Wei is a cosmopolitan fungal pathogen with a broad host range, infecting numerous economic and ornamental plants such as *Jasminum nudiflorum* [2], *Strobilanthes cusia* [3], tobacco [4], and *Syringa spp.*[5]. However, there is no previous report of C. cassiicola infecting B. fimbristipula.

In this study, we conducted field surveys, pathogen isolation, morphological and molecular identification, and pathogenicity assays to confirm the causal agent of B. fimbristipula leaf spot. This work is the first to report C. cassiicola as a pathogen of B. fimbristipula in China, and provides essential information for accurate disease diagnosis and the development of effective management strategies.

## Materials and methods

### Field survey and sample collection

Field surveys were conducted in August 2024 in Huangping County (27.020840°N, 107.799035°E), Guizhou, and Guzhang County (28.600193°N, 109.936298°E), Hunan, China. Disease incidence, symptom progression, and yield loss were recorded. For pathogen isolation, 50 symptomatic leaves with typical lesions were randomly collected, and samples were excised from the healthy-diseased interface to ensure pathogen viability.

### Pathogen isolation and morphological identification

Diseased leaf tissues (5 mm²) from the lesion margin were surface-sterilized with 75% ethanol for 45 s, rinsed five times with sterile distilled water, and dried on sterile filter paper. The sterilized tissues were placed on potato dextrose agar (PDA) medium and incubated at 25 ± 1°C in the dark for 7 days. Pure cultures were obtained by single-spore isolation [6], and 10 representative isolates (ZBTK1–ZBTK10) were selected from 121 recovered isolates for further characterization.

Colony morphology (color, texture, aerial hyphae) was observed and recorded after 7 days of incubation. Conidial characteristics (shape, color, size, pseudoseptum number) were examined under a light microscope (Olympus BX53, Japan). A total of 50 conidia were measured to obtain the size range and mean ± standard deviation (SD). Morphological identification was performed by comparing the characteristics with known descriptions of *Corynespora cassiicola* [7].

Permits and permissions: No specific permits were required for the collection of diseased *Begonia fimbristipula* leaves in Huangping County (Guizhou) and Guzhang County (Hunan), as the plants are cultivated medicinal species and sampling was carried out on agricultural land with landowner consent. The study did not involve endangered or protected species.

### Molecular identification

#### Genomic DNA extraction.

Isolate ZBTK-1 (randomly selected as the representative isolate) was cultured on PDA for 8 days, and fresh mycelia were collected for genomic DNA extraction using the Solarbio DNA Extraction Kit (Solarbio Biotech Co., Beijing, China) following the manufacturer’s instructions. The concentration and purity of extracted DNA were detected by a NanoDrop 2000 spectrophotometer (Thermo Fisher Scientific, USA).

#### PCR amplification and sequencing.

Three molecular markers were amplified by PCR: internal transcribed spacer (ITS), translation elongation factor 1-alpha (TEF1α), and RNA polymerase II subunit (RPB2). The specific PCR primers were as follows: ITS1/ITS4 for ITS [8], EF1-728F/EF1-986R for TEF1α [9], and RPB2-5F/RPB2-7cR for RPB2 [10].

PCR reactions were performed in a 25 μL volume containing 12.5 μL 2 × Taq PCR Master Mix (Tiangen Biotech Co., Beijing, China), 1 μL of each forward and reverse primer (10 μM), 2 μL genomic DNA (50 ng/μL), and 8.5 μL sterile ddH₂O. The PCR program for ITS was: 94°C pre-denaturation for 5 min; 35 cycles of 94°C denaturation for 30 s, 55°C annealing for 30 s, 72°C extension for 1 min; and 72°C final extension for 10 min. The annealing temperatures for TEF1α and RPB2 were 58°C and 60°C, respectively, with the same other PCR conditions.

PCR products were detected by 1% agarose gel electrophoresis and sequenced by Tsingke Biotech Co., Ltd. (Changsha, China). The obtained sequences were submitted to the GenBank database, with accession numbers PQ899537 (ITS), PV549328 (TEF1α), and PV549329 (RPB2).

#### Phylogenetic analysis.

The obtained ITS, TEF1α, and RPB2 sequences were concatenated using BioEdit 7.2.5. Phylogenetic analysis was performed using MEGA 11 software with the Maximum Likelihood (ML) method and 5000 bootstrap replicates. The Kimura 2-parameter (K2P) model was used for distance calculation. Known C. cassiicola sequences from GenBank were included as references for phylogenetic clustering analysis.

#### Pathogenicity assays.

Pathogenicity was verified by artificial inoculation on 30-day-old healthy B. fimbristipula seedlings. Mycelial plugs (5 mm in diameter) were cut from the edge of 8-day-old PDA cultures of the 10 isolates (ZBTK1–ZBTK10). The adaxial surfaces of healthy leaves were wounded with a sterile needle and inoculated with mycelial plugs (one plug per leaf). Seedlings inoculated with sterile PDA plugs were used as the control.

All inoculated seedlings were maintained in a greenhouse under controlled conditions: 25 ± 2°C, 90% relative humidity, and a 12-h light/dark photoperiod. Each isolate was applied to five independent plants with five leaves inoculated per plant, and the experiment was repeated three times. Disease symptoms were observed and recorded daily for 14 days post-inoculation (dpi).

Disease development was evaluated qualitatively by visual inspection of symptom appearance, consistent with standard practice for first report studies. To fulfill Koch’s postulates, the pathogen was re-isolated from the diseased leaves of inoculated seedlings using the same tissue culture method described above. The re-isolated pathogen was identified by morphological characteristics and molecular sequencing to confirm consistency with the original isolate ZBTK-1.

## Results

### Field disease symptoms and incidence

Severe leaf spot disease outbreaks were observed on B. fimbristipula in Guizhou and Hunan Provinces, with a disease incidence of over 60% across 667 hectares of cultivation area and a fresh leaf yield loss of more than 20%. The initial disease symptoms were small dark-brown circular lesions (2–5 mm in diameter) with yellow halos on the leaves. As the disease progressed, the lesions expanded, coalesced into irregular or semicircular necrotic areas (15–30 mm in diameter), and eventually caused leaf yellowing and abscission (defoliation) of infected plants.

### Morphological characteristics of the isolate

After 7 days of incubation on PDA at 25 ± 1°C, all 10 representative isolates formed gray to dark-gray colonies with a smooth surface and sparse aerial hyphae. Conidia were pale-brown to olivaceous, straight or slightly curved, solitary or in unbranched chains, with a rounded base and a pointed apex. Conidial size ranged from 32.3–132.8 × 5.8–8.4 µm (mean ± SD: 81.4 ± 12.6 × 6.3 ± 0.4 µm, n = 50), with 0–15 pseudosepta. These morphological characteristics were consistent with the taxonomic description of Corynespora cassiicola [6].

### Molecular phylogenetic analysis

The ITS (538 bp), TEF1α (302 bp), and RPB2 (1192 bp) sequences of isolate ZBTK-1 were successfully amplified and sequenced. Phylogenetic analysis based on the concatenated ITS-TEF1α-RPB2 sequences showed that ZBTK-1 clustered within the C. cassiicola clade with >98% bootstrap support, forming a monophyletic group with known C. cassiicola reference strains from GenBank. Combined with morphological identification, the isolate was confirmed as *Corynespora cassiicola*.

### Pathogenicity test results

Typical leaf spot symptoms (dark-brown lesions with yellow halos) were observed on the inoculated B. fimbristipula leaves at 7 dpi, and the symptoms were consistent with those observed in the field. As the incubation time increased, the lesions expanded and coalesced, leading to leaf necrosis. No disease symptoms were observed on the control leaves inoculated with sterile PDA plugs throughout the experiment.

The pathogen was successfully re-isolated from the diseased tissues of inoculated seedlings, and the re-isolated fungus had the same morphological characteristics and molecular sequences as the original isolate ZBTK-1. The pathogenicity assays were repeated three times with consistent results, fulfilling Koch’s postulates and confirming that C. cassiicola is the causal agent of B. fimbristipula leaf spot.

## Discussion

This study is the first report of *Corynespora cassiicola* causing leaf spot on Begonia fimbristipula in China, expanding the known host range of this cosmopolitan fungal pathogen. Prior to this study, C. cassiicola has been reported to infect a variety of economic plants in China, including *Jasminum nudiflorum* [2], *Strobilanthes cusia* [3], tobacco [4], and *Syringa* spp. [5], but there was no record of its infection on Begoniaceae plants such as B. fimbristipula. Our findings enrich the host database of C. cassiicola and provide a new reference for the study of the host range and pathogenic evolution of this pathogen.

**Fig 1:**
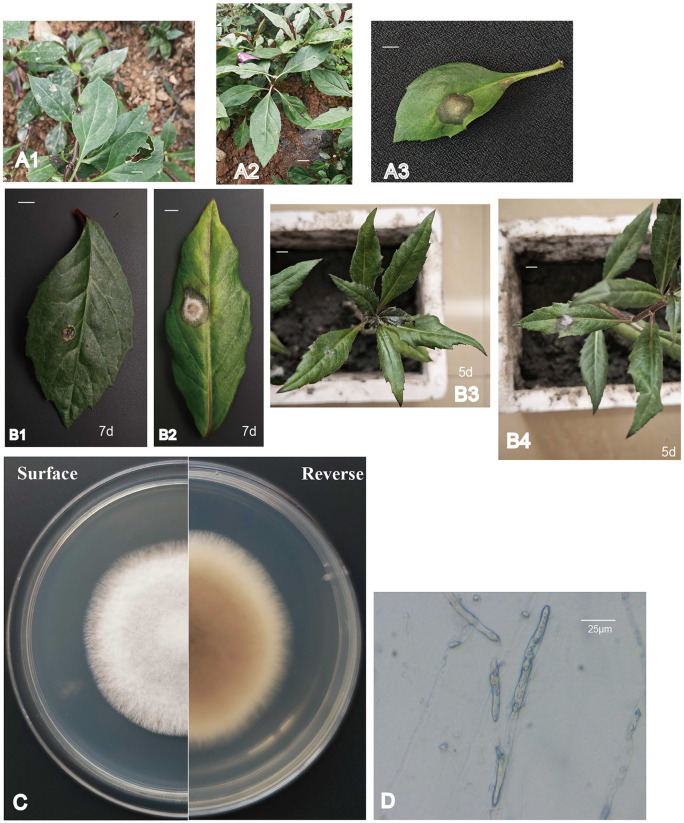
Leaf spot of *Begonia fimbristipula* caused by *Corynespora cassiicola.* (A1–A3) Field symptoms on naturally infected leaves in Guizhou and Hunan, China: (A1) early stage with small dark-brown circular lesions and yellow halos; (A2) progressive stage with expanding and coalescing lesions; (A3) severe stage with leaf yellowing and necrosis. (B1–B2) Pathogenicity assay – leaf detail: (B1) control leaf inoculated with sterile PDA plug (no symptoms); (B2) leaf inoculated with C. cassiicola mycelial plug showing typical lesions at 14 days post-inoculation. (B3–B4) Pathogenicity assay – whole plant view: (B3) control plant (no symptoms); (B4) inoculated plant showing symptom development. (C) Colony morphology on PDA after 7 days at 25°C: left – surface view; right – reverse view. (D) Conidia of isolate ZBTK-1, pale-brown to olivaceous, straight or slightly curved, with 0–15 pseudosepta. Scale bar = 20 μm.

**Fig 2:**
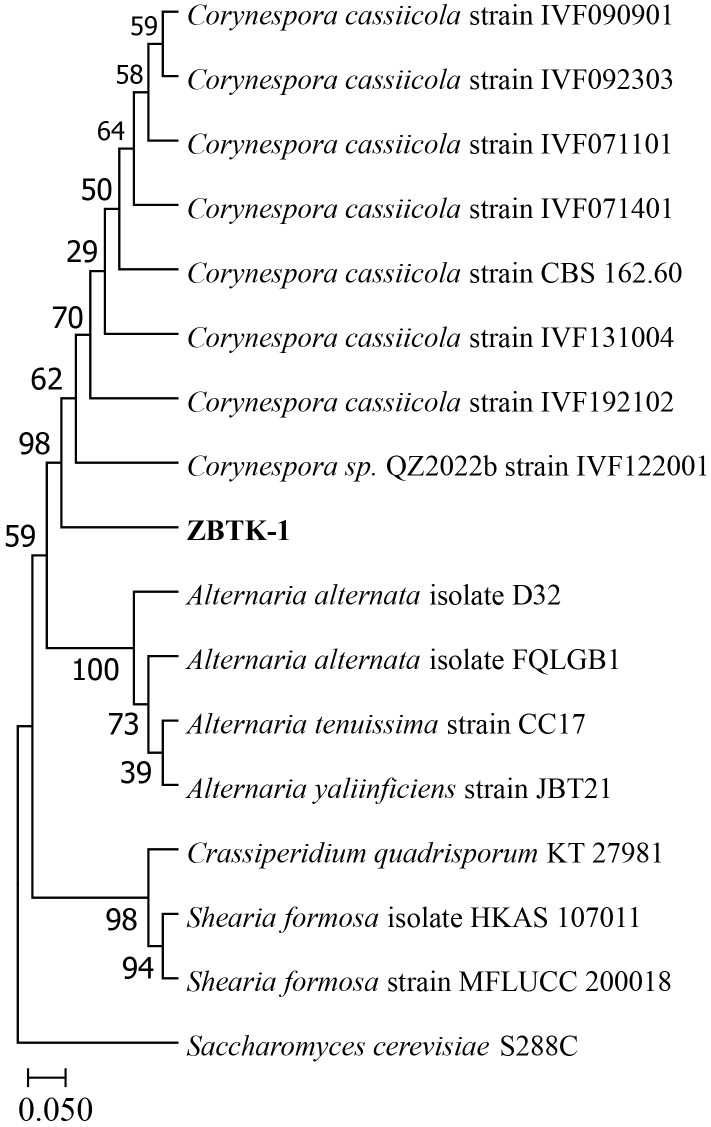
Phylogenetic tree based on concatenated ITS, TEF1α, and RPB2 sequences. The tree was constructed using the neighbor-joining (NJ) method with 1000 bootstrap replicates in MEGA 11. Bootstrap support values (≥50%) are shown at branch nodes. Isolate ZBTK-1 (in bold) clusters within the *Corynespora cassiicola* clade.

B. fimbristipula is an important medicinal plant in southern China, and the newly emerging leaf spot disease caused by C. cassiicola has led to severe yield losses (≥20%) in Guizhou and Hunan Provinces Fig 1. The wide host range and strong adaptability of C. cassiicola increase the risk of disease spread to other Begonia species and adjacent crops, posing a potential threat to the local medicinal plant and agricultural production. Therefore, it is urgent to develop effective integrated disease management strategies for this disease Fig 2.

Based on our findings and the biological characteristics of C. cassiicola, we suggest that future research should evaluate integrated management strategies. Potential approaches include: (1) Cultural control – removing and incinerating diseased leaves to reduce inoculum, and improving field ventilation and drainage to lower humidity; (2) Chemical control – screening effective fungicides (e.g., azoles, strobilurins) for field application at early disease stages; and (3) Resistance breeding – evaluating germplasm resources of B. fimbristipula to identify resistant varieties. These strategies have not been experimentally tested in this study and remain to be validated.

This study has some limitations: we only identified the causal pathogen and verified its pathogenicity, but did not investigate the pathogenic mechanism of C. cassiicola on B. fimbristipula (e.g., effector proteins, cell wall-degrading enzymes) or the optimal environmental conditions for disease occurrence (e.g., temperature, humidity, rainfall). Future studies will focus on these aspects to clarify the disease epidemiology and develop more precise control strategies. In addition, the composite infection of C. cassiicola with other pathogens on B. fimbristipula also needs to be further investigated, as composite infections are common in field conditions and may exacerbate disease severity. Additionally, pathogenicity was assessed qualitatively based on symptom presence/absence and re-isolation, which is standard for first reports. Quantitative disease severity metrics (e.g., lesion size, disease index) and statistical comparisons were not performed, and we acknowledge this as a limitation.

In conclusion, our study confirmed *Corynespora cassiicola* as the causal agent of Begonia fimbristipula leaf spot in China, providing a critical basis for the diagnosis of this disease and for future studies on its epidemiology and control. This work also highlights the need for increased attention to the emerging diseases of medicinal plants and the importance of timely pathogen identification for effective disease control.
