# Supplementary material for: First report of Corynespora cassiicola causing leaf spot on Begonia fimbristipula in China
Source: PLoS One. 2026 Jul 30;21(7):e0346996. doi: 10.1371/journal.pone.0346996 (PMC13422844; doi:10.1371/journal.pone.0346996)
Supplement: Attachment — Submitted filename: Response to Reviewers.docx [file pone.0346996.s003.docx]

**Response to Reviewers**

Manuscript ID: PONE-D-26-14713
Title: First Report of Corynespora cassiicola Causing Leaf Spot on Begonia fimbristipula in China

Dear Editor and Reviewers,

We thank the Academic Editor and the three reviewers for their careful reading and constructive comments. We have revised the manuscript accordingly. Please note that this is a First Report study, and we have not conducted additional experiments or collected new data. Instead, we have clarified the qualitative nature of our pathogenicity assessment, toned down overstatements, added explicit limitations, and corrected errors. Below we address each point.

Responses to Reviewer #1

Comment 1: Language and grammar issues.
Response: The entire manuscript has been edited for clarity. The PCR program description has been streamlined, and the duplicate section removed. Figure captions rewritten.

Comment 2: Low-resolution figures; contradiction in phylogenetic method.
Response: We have replaced the figures with the best resolution attainable from our original image files. The method is now consistently described as “neighbor-joining (NJ) with 1000 bootstrap replicates” in both text and figure caption.

Comment 3: Yield loss estimation method not described.
Response: Added: “Yield loss was estimated by field observation comparing symptomatic vs. asymptomatic plants in representative plots.”

Comment 4: Clarify whether all 10 isolates were sequenced.
Response: Clarified in Methods: “Only ZBTK-1 was used for multilocus sequencing because ITS sequences of all 10 isolates were 100% identical (data not shown).” Also noted as a limitation in Discussion.

Comment 5 (suggested citation): Added as requested.

Responses to Reviewer #2

Reviewer #2 raised many short points. We address the main categories:

Missing methodological details (survey duration, incidence calculation, sterilization justification, isolate selection, plant age): Added to Methods using existing records. No new experiments.

Statistical analysis: We state that pathogenicity was assessed qualitatively, which is standard for first reports. No statistical tests were performed. A limitation statement added.

Redundancy: Duplicate section removed.

“±0°C” : Corrected to “25 ± 1°C”.

Responses to Reviewer #3

Comment 1 (quantitative pathogenicity data and statistics):
We thank the reviewer for the suggestion. However, in the context of a First Report, the standard requirement for pathogenicity confirmation is qualitative fulfillment of Koch’s postulates – i.e., symptom reproduction and re-isolation – without mandatory quantitative metrics or statistical analysis. This is consistent with countless First Reports published in Plant Disease, Journal of Plant Pathology, and other journals, including previous reports of C. cassiicola on new hosts (e.g., references [2–5] in our manuscript). Therefore, in keeping with standard practice for First Reports, we did not perform quantitative lesion measurements. We have, however, explicitly acknowledged this as a limitation in the Discussion and clarified in Methods that assessment was qualitative. We hope the reviewer will accept this as sufficient for a first report.

Comment 2 (single isolate for multilocus sequencing):
We acknowledge the limitation. We have clarified that ITS sequences of all 10 isolates were identical, and that only one isolate was fully sequenced due to resource constraints. A limitation statement added in Discussion.

Comment 3 (duplicated Methods):
Deleted.

Comment 4 (statistical analysis minimal):
As noted above, the study is descriptive; no inferential statistics were performed. We have stated this explicitly in Methods and Discussion.

Comment 5 (data availability):
Data Availability Statement updated. All sequence data are in GenBank. Morphological data (conidial dimensions) are reported in Results. No other raw data were collected.

Comment 6 (overstatements in Discussion/Conclusion):
We have significantly moderated the language. Management suggestions are now presented as future research directions, not as demonstrated outcomes. Conclusion revised to remove claims about integrated management.

Comment 7 (English language):
Manuscript has been carefully edited.

Journal Requirements

#2 Permits: Added to Methods.
#3 Data Availability: Updated in submission system.
#4 Role of funders: Added to cover letter.
#5 LaTeX template: We have followed PLOS ONE’s formatting guidelines as closely as possible. Regarding the LaTeX template requirement, we would like to request permission to submit in Word format, as our manuscript was originally prepared in Word and conversion would introduce formatting errors.
#6, #7 Figure 1: Added, cited in Results, caption provided.

We believe the revised manuscript meets the standards for a First Report and respectfully request acceptance.

Sincerely,
Xun Lu
