# Supplementary material for: First report of Corynespora cassiicola causing leaf spot on Begonia fimbristipula in China
Source: PLoS One. 2026 Jul 30;21(7):e0346996. doi: 10.1371/journal.pone.0346996 (PMC13422844; doi:10.1371/journal.pone.0346996)
Supplement: Attachment — Submitted filename: Reviewed File.docx [file pone.0346996.s001.docx]

**First Report of *Corynespora cassiicola* Causing Leaf Spot on *Begonia fimbristipula* in China**

**Xiaobo Zhang,^1^ Xiaoyi Zuo,^2^ Jianhua Lu,^2^ Ya Xiao,^2^ Yuanshun Wang,^2^ Huan Chen,^2^ Yuting Zhang,^3^ and Xun Lu^2,†^**

^1^Kaili University, Kaili 556011, Guizhou Province, People’s Republic of China

^2^Agricultural Science Institute of Xiangxi Tujia and Miao Autonomous Prefecture, Jishou 416000

^3^Public Resource Centre of XiangXi Tujia and Miao Autonomous Prefecture, 416000

Hunan Province, People’s Republic of China

Corresponding author: Xun Lu; E-mail:280923101@qq.com

**Abstract**

**Background:** *Begonia fimbristipula* Hance is an economically important perennial medicinal plant in southern China, with anti-inflammatory and respiratory therapeutic properties. Leaf spot disease has recently caused severe yield losses to *B. fimbristipula* in Guizhou and Hunan Provinces, but the causal pathogen remains unknown.

**Methods:** Field surveys were conducted to record disease symptoms and incidence. The pathogen was isolated from diseased leaves via tissue culture, and identified by combining morphological characteristics and multilocus phylogenetic analysis (ITS, TEF1α, RPB2). Pathogenicity was verified by fulfilling Koch’s postulates through artificial inoculation and re-isolation assays.

**Results:** Severe leaf spot outbreaks were observed in August 2024, with over 60% disease incidence across 667 hectares and >20% fresh leaf yield loss. The isolated pathogen was identified as Corynespora cassiicola based on gray to dark-gray colonies, pale-brown olivaceous conidia with 0–15 pseudosepta, and ≥98% bootstrap support in phylogenetic clustering with known *C. cassiicola* strains. Artificial inoculation induced typical field leaf spot symptoms on *B. fimbristipula* seedlings, and the pathogen was successfully re-isolated from diseased tissues.

**Conclusions:** This is the first report of *C. cassiicola* causing leaf spot on *B. fimbristipula* in China, expanding the host range of this pathogen. Our findings provide a critical basis for the diagnosis and integrated management of this newly emerging disease on the medicinal plant.

**Keywords:** *Corynespora cassiicola*; *Begonia fimbristipula*; leaf spot; medicinal plant; phylogenetic analysis; pathogenicity

**Introduction**

*Begonia fimbristipula* Hance (Zibeitiankui, a medicinal begonia), a perennial herb belonging to the Begoniaceae family, is widely distributed in southern China and valued for its medicinal properties, including anti-inflammatory and respiratory disease treatment effects^[1]^. In recent years, the large-scale cultivation of *B. fimbristipula* has been promoted for its economic and medicinal value, but plant diseases have become a major limiting factor for its production.

In August 2024, a severe leaf spot disease was observed on *B. fimbristipula* in Huangping County (Guizhou Province) and Guzhang County (Hunan Province), leading to extensive defoliation and significant yield loss. *Corynespora cassiicola* (Berk. & M.A. Curtis) C.T. Wei is a cosmopolitan fungal pathogen with a broad host range, infecting numerous economic and ornamental plants such as *Jasminum nudiflorum*^[2]^, *Strobilanthes cusia*^[3]^, tobacco^[4]^, and *Syringa spp.*^[5]^. However, there is no previous report of *C. cassiicola* infecting *B. fimbristipula*.

In this study, we conducted field surveys, pathogen isolation, morphological and molecular identification, and pathogenicity assays to confirm the causal agent of *B. fimbristipula* leaf spot. This work is the first to report *C. cassiicola* as a pathogen of *B. fimbristipula* in China, and provides essential information for disease diagnosis and control of this medicinal plant.

**Materials and Methods**

**Field Survey and Sample Collection**

Field surveys were carried out in August 2024 in Huangping County (27.020840°N, 107.799035°E), Guizhou Province and Guzhang County (28.600193°N, 109.936298°E), Hunan Province, China. Disease incidence, symptom development, and yield loss of *B. fimbristipula* were recorded. A total of 50 symptomatic leaves with typical leaf spot symptoms were randomly collected for pathogen isolation, with all samples taken from the healthy-diseased interface of lesions to ensure pathogen viability.

**Pathogen Isolation and Morphological Identification**

Diseased leaf tissues (5 mm²) from the lesion margin were surface-sterilized with 75% ethanol for 45 s, rinsed five times with sterile distilled water, and dried on sterile filter paper. The sterilized tissues were placed on potato dextrose agar (PDA) medium and incubated at 25±0°C in the dark for 7 days. Pure cultures were obtained by single-spore isolation, and 10 representative isolates (ZBTK1–ZBTK10) were selected from 121 recovered isolates for further characterization.

Colony morphology (color, texture, aerial hyphae) was observed and recorded after 7 days of incubation. Conidial characteristics (shape, color, size, pseudoseptum number) were examined under a light microscope (Olympus BX53, Japan). A total of 50 conidia were measured to obtain the size range and mean ± standard deviation (SD). Morphological identification was performed by comparing the characteristics with known descriptions of *Corynespora cassiicola* ^[6]^.

**Molecular Identification**

**Genomic DNA Extraction**

Isolate ZBTK-1 (randomly selected as the representative isolate) was cultured on PDA for 8 days, and fresh mycelia were collected for genomic DNA extraction using the Solarbio DNA Extraction Kit (Solarbio Biotech Co., Beijing, China) following the manufacturer’s instructions. The concentration and purity of extracted DNA were detected by a NanoDrop 2000 spectrophotometer (Thermo Fisher Scientific, USA).

**PCR Amplification and Sequencing**

Three molecular markers were amplified by PCR: internal transcribed spacer (ITS), translation elongation factor 1-alpha (TEF1α), and RNA polymerase II subunit (RPB2). The specific PCR primers were as follows: ITS1/ITS4 for ITS^[7]^, EF1-728F/EF1-986R for TEF1α^[8]^, and RPB2-5F/RPB2-7cR for RPB2^[9]^.

PCR reactions were performed in a 25 μL volume containing 12.5 μL 2× Taq PCR Master Mix (Tiangen Biotech Co., Beijing, China), 1 μL of each forward and reverse primer (10 μM), 2 μL genomic DNA (50 ng/μL), and 8.5 μL sterile ddH₂O. The PCR program for ITS was: 94°C pre-denaturation for 5 min; 35 cycles of 94°C denaturation for 30 s, 55°C annealing for 30 s, 72°C extension for 1 min; and 72°C final extension for 10 min. The annealing temperatures for TEF1α and RPB2 were 58°C and 60°C, respectively, with the same other PCR conditions.

PCR products were detected by 1% agarose gel electrophoresis and sequenced by Tsingke Biotech Co., Ltd. (Changsha, China). The obtained sequences were submitted to the GenBank database, with accession numbers PQ899537 (ITS), PV549328 (TEF1α), and PV549329 (RPB2).

**Phylogenetic Analysis**

The obtained ITS, TEF1α, and RPB2 sequences were concatenated using BioEdit 7.2.5. Phylogenetic analysis was performed using MEGA 11 software with the neighbor-joining (NJ) method and 1000 bootstrap replicates. The Kimura 2-parameter (K2P) model was used for distance calculation. Known *C. cassiicola* sequences from GenBank were included as references for phylogenetic clustering analysis.

**Pathogenicity Assays**

Pathogenicity was verified by artificial inoculation on 30-day-old healthy *B. fimbristipula* seedlings. Mycelial plugs (5 mm in diameter) were cut from the edge of 8-day-old PDA cultures of the 10 isolates (ZBTK1–ZBTK10). The adaxial surfaces of healthy leaves were wounded with a sterile needle and inoculated with mycelial plugs (one plug per leaf). Seedlings inoculated with sterile PDA plugs were used as the control.

All inoculated seedlings were maintained in a greenhouse under controlled conditions: 25±2°C, 90% relative humidity, and a 12-h light/dark photoperiod. Each isolate was applied to five independent plants with five leaves inoculated per plant, and the experiment was repeated three times. Disease symptoms were observed and recorded daily for 14 days post-inoculation (dpi).

To fulfill Koch’s postulates, the pathogen was re-isolated from the diseased leaves of inoculated seedlings using the same tissue culture method described above. The re-isolated pathogen was identified by morphological characteristics and molecular sequencing to confirm consistency with the original isolate ZBTK-1.

### Molecular Identification

#### Genomic DNA Extraction

Isolate ZBTK-1 (randomly selected as the representative isolate) was cultured on PDA for 8 days, and fresh mycelia were collected for genomic DNA extraction using the Solarbio DNA Extraction Kit (Solarbio Biotech Co., Beijing, China) following the manufacturer’s instructions. The concentration and purity of extracted DNA were detected by a NanoDrop 2000 spectrophotometer (Thermo Fisher Scientific, USA).

#### PCR Amplification and Sequencing

Three molecular markers were amplified by PCR: internal transcribed spacer (ITS), translation elongation factor 1-alpha (TEF1α), and RNA polymerase II subunit (RPB2). The specific PCR primers were as follows: ITS1/ITS4 for ITS^[7]^, EF1-728F/EF1-986R for TEF1α^[8]^, and RPB2-5F/RPB2-7cR for RPB2^[9]^.

PCR reactions were performed in a 25 μL volume containing 12.5 μL 2× Taq PCR Master Mix (Tiangen Biotech Co., Beijing, China), 1 μL of each forward and reverse primer (10 μM), 2 μL genomic DNA (50 ng/μL), and 8.5 μL sterile ddH₂O. The PCR program for ITS was: 94°C pre-denaturation for 5 min; 35 cycles of 94°C denaturation for 30 s, 55°C annealing for 30 s, 72°C extension for 1 min; and 72°C final extension for 10 min. The annealing temperatures for TEF1α and RPB2 were 58°C and 60°C, respectively, with the same other PCR conditions.

PCR products were detected by 1% agarose gel electrophoresis and sequenced by Tsingke Biotech Co., Ltd. (Changsha, China). The obtained sequences were submitted to the GenBank database, with accession numbers PQ899537 (ITS), PV549328 (TEF1α), and PV549329 (RPB2).

#### Phylogenetic Analysis

The obtained ITS, TEF1α, and RPB2 sequences were concatenated using BioEdit 7.2.5. Phylogenetic analysis was performed using MEGA 11 software with the **neighbor-joining (NJ)** method and **1000 bootstrap replicates**. The Kimura 2-parameter (K2P) model was used for distance calculation. Known C. cassiicola sequences from GenBank were included as references for phylogenetic clustering analysis.

### Pathogenicity Assays

Pathogenicity was verified by artificial inoculation on 30-day-old healthy B. fimbristipula seedlings. Mycelial plugs (5 mm in diameter) were cut from the edge of 8-day-old PDA cultures of the 10 isolates (ZBTK1–ZBTK10). The adaxial surfaces of healthy leaves were inoculated with mycelial plugs (one plug per leaf). Seedlings inoculated with sterile PDA plugs were used as the control.

All inoculated seedlings were maintained in a greenhouse under controlled conditions: 25±2°C, 90% relative humidity, and a 12-h light/dark photoperiod. Each isolate was applied to five independent plants with five leaves inoculated per plant, and the experiment was repeated three times. Disease symptoms were observed and recorded daily for 14 days post-inoculation (dpi).

To fulfill Koch’s postulates, the pathogen was re-isolated from the diseased leaves of inoculated seedlings using the same tissue culture method described above. The re-isolated pathogen was identified by morphological characteristics and molecular sequencing to confirm consistency with the original isolate ZBTK-1.

## Results

### Field Disease Symptoms and Incidence

Severe leaf spot disease outbreaks were observed on B. fimbristipula in Guizhou and Hunan Provinces, with a disease incidence of over 60% across 667 hectares of cultivation area and a fresh leaf yield loss of more than 20%. The initial disease symptoms were small dark-brown circular lesions (2–5 mm in diameter) with yellow halos on the leaves. As the disease progressed, the lesions expanded, coalesced into irregular or semicircular necrotic areas (15–30 mm in diameter), and eventually caused leaf yellowing and abscission (defoliation) of infected plants.

### Morphological Characteristics of the Isolate

After 7 days of incubation on PDA at 25±0°C, all 10 representative isolates formed **gray to dark-gray colonies** with a smooth surface and sparse aerial hyphae. Conidia were **pale-brown to olivaceous**, straight or slightly curved, solitary or in unbranched chains, with a rounded base and a pointed apex. Conidial size ranged from 32.3–132.8 × 5.8–8.4 µm (mean ± SD: 81.4±12.6 × 6.3±0.4 µm, n=50), with 0–15 pseudosepta. These morphological characteristics were consistent with the taxonomic description of Corynespora cassiicola^[6]^.

### Molecular Phylogenetic Analysis

The ITS (538 bp), TEF1α (302 bp), and RPB2 (1192 bp) sequences of isolate ZBTK-1 were successfully amplified and sequenced. Phylogenetic analysis based on the concatenated ITS-TEF1α-RPB2 sequences showed that ZBTK-1 **clustered within the C. cassiicola clade with >98% bootstrap support**, forming a monophyletic group with known C. cassiicola reference strains from GenBank. Combined with morphological identification, the isolate was confirmed as Corynespora cassiicola.

### Pathogenicity Test Results

Typical leaf spot symptoms (dark-brown lesions with yellow halos) were observed on the inoculated B. fimbristipula leaves at **7 dpi**, and the symptoms were consistent with those observed in the field. As the incubation time increased, the lesions expanded and coalesced, leading to leaf necrosis. No disease symptoms were observed on the control leaves inoculated with sterile PDA plugs throughout the experiment.

The pathogen was successfully re-isolated from the diseased tissues of inoculated seedlings, and the re-isolated fungus had the same morphological characteristics and molecular sequences as the original isolate ZBTK-1. The pathogenicity assays were repeated three times with consistent results, **fulfilling Koch’s postulates** and confirming that C. cassiicola is the causal agent of B. fimbristipula leaf spot.

## Discussion

This study is the **first report of Corynespora cassiicola causing leaf spot on Begonia fimbristipula in China**, expanding the known host range of this cosmopolitan fungal pathogen. Prior to this study, C. cassiicola has been reported to infect a variety of economic plants in China, including Jasminum nudiflorum^[2]^, Strobilanthes cusia^[3]^, tobacco^[4]^, and Syringa spp.^[5]^, but there was no record of its infection on Begoniaceae plants such as B. fimbristipula. Our findings enrich the host database of C. cassiicola and provide a new reference for the study of the host range and pathogenic evolution of this pathogen.

B.fimbristipula is an important medicinal plant in southern China, and the newly emerging leaf spot disease caused by C. cassiicola has led to severe yield losses (≥20%) in Guizhou and Hunan Provinces. The wide host range and strong adaptability of C. cassiicola increase the risk of disease spread to other Begonia species and adjacent crops, posing a potential threat to the local medicinal plant and agricultural production. Therefore, it is urgent to develop effective integrated disease management strategies for this disease.

Based on our findings and the biological characteristics of C. cassiicola, targeted control measures should be implemented: (1) **Cultural control**: Improve field sanitation by removing and incinerating diseased leaves and plants to reduce the initial inoculum; optimize field ventilation and drainage to reduce relative humidity and inhibit pathogen infection. (2) **Chemical control**: Conduct fungicide screening trials to select effective bactericides (e.g., azoles, strobilurins) for field application, and apply them at the initial disease stage to control disease spread. (3) **Resistance breeding**: Conduct resistance evaluation of different B. fimbristipula germplasm resources to breed disease-resistant varieties, which is the most economical and effective long-term control measure.

This study has some limitations: we only identified the causal pathogen and verified its pathogenicity, but did not investigate the pathogenic mechanism of C. cassiicola on B. fimbristipula (e.g., effector proteins, cell wall-degrading enzymes) or the optimal environmental conditions for disease occurrence (e.g., temperature, humidity, rainfall). Future studies will focus on these aspects to clarify the disease epidemiology and develop more precise control strategies. In addition, the composite infection of C. cassiicola with other pathogens on B. fimbristipula also needs to be further investigated, as composite infections are common in field conditions and may exacerbate disease severity.

In conclusion, our study confirmed Corynespora cassiicola as the causal agent of Begonia fimbristipula leaf spot in China, providing a critical basis for the diagnosis, monitoring, and integrated management of this disease. This work also highlights the need for increased attention to the emerging diseases of medicinal plants and the importance of timely pathogen identification for effective disease control.

## Funding

This work was supported by the "Doctor Professor Service Group" Special Project of Kaili University (2024, No. BS20240214); the Integrated Research Team for Resource Utilization and Fauna & Flora Conservation of Kaili University (2025, No. YTH-TD20255I); the Natural Science Research Youth Project of the Department of Education of Guizhou Province (2024, No. Qian Jiaoji [2024]230); and Youth Science and Technology Innovation Fund(XXNK202543). The funders had no role in study design, data collection and analysis, decision to publish, or preparation of the manuscript.

## Author Contributions

- **Conceptualization**: Xun Lu, Xiaobo Zhang
- **Methodology**: Xun Lu, Xiaoyi Zuo,Yuanshun Wang
- **Investigation**: Hua Quan, Jianhua Lu, Ya Xiao, Huan Chen, Yuting Zhang
- **Data Curation**: Xiaobo Zhang, Xiaoyi Zuo
- **Formal Analysis**: Xiaobo Zhang
- **Writing – Original Draft**: Xiaobo Zhang
- **Writing – Review & Editing**: Xun Lu
- **Supervision**: Xun Lu
- **Funding Acquisition**: Xun Lu，Xiaobo Zhang

## Conflict of Interest Statement

The authors declare that the research was conducted in the absence of any commercial or financial relationships that could be construed as a potential conflict of interest.

## Acknowledgments

We thank the staff of the Agricultural Science Institute of Xiangxi Tujia and Miao Autonomous Prefecture for their assistance in field surveys and sample collection.

**References**

[1] Han L, Li Y, Zhang H, et al. Antioxidant and anti-inflammatory activities of extracts from Begonia fimbristipula Hance. Oxid Antioxid Med Sci.2013; 2(3):195–200.

[2] Hu YF, Wang Y, Li J, et al. First report of Corynespora cassiicola causing leaf spot on Jasminum nudiflorum in China. Plant Dis.2023; 107:2521.

[3] Liu BC, Zhang L, Wang X, et al. First report of Corynespora cassiicola causing leaf spot on Strobilanthes cusia in China. Plant Dis.2023; 107:561.

[4] Lan D, Wu Y, Chen X, et al. Characterization of Corynespora cassiicola isolates causing leaf spot on tobacco in southern China. Trop Plant Pathol.2025; 50:34. <https://doi.org/10.1007/s40858-024-00968-9>

[5] Xie SF, Wang C, Zhang Y, et al. First report of Corynespora cassiicola causing leaf spot on Syringa spp. in China. Plant Dis.2021; 105:509.

[6] Wang S, Li Y, Zhang Q, et al. First report of Corynespora cassiicola causing leaf spot on Acanthus ilicifolius in China. For Pathol.2021; 51:e12684.

[7] White TJ, Bruns T, Lee S, Taylor J. Amplification and direct sequencing of fungal ribosomal RNA genes for phylogenetics. In: PCR Protocols: A Guide to Methods and Applications, San Diego: Academic Press;1990.p. 315-322.

[8] Carbone I, Kohn LM. A method for designing primer sets for speciation studies in filamentous ascomycetes. Mycologia.1999; 91(3):553–556.

[9] Liu YJ, Rehner SA, Buckley E. Phylogenetic relationships among ascomycetes: evidence from an RNA polymerase II subunit. Mol Phylogenet Evol.1999; 12(3):416–424.
