# Supplementary material for: First report of Corynespora cassiicola causing leaf spot on Begonia fimbristipula in China
Source: PLoS One. 2026 Jul 30;21(7):e0346996. doi: 10.1371/journal.pone.0346996 (PMC13422844; doi:10.1371/journal.pone.0346996)
Supplement: Peer review history

# Peer review: Decision Letter 0

**DOI:** 10.1371/journal.pone.0346996.r001

**Authors:** Raed Haleem

23 Apr 2026

PONE-D-26-14713

First Report of Corynespora cassiicola Causing Leaf Spot on Begonia fimbristipula in China

PLOS One

Dear Dr. Lu,

Thank you for submitting your manuscript to PLOS ONE. After careful consideration, we feel that it has merit but does not fully meet PLOS ONE’s publication criteria as it currently stands. Therefore, we invite you to submit a revised version of the manuscript that addresses the points raised during the review process.

Please submit your revised manuscript by Jun 06 2026 11:59PM. If you will need more time than this to complete your revisions, please reply to this message or contact the journal office at plosone@plos.org. When you're ready to submit your revision, log on to https://www.editorialmanager.com/pone/ and select the 'Submissions Needing Revision' folder to locate your manuscript file.

Please include the following items when submitting your revised manuscript:

- A letter that responds to each point raised by the academic editor and reviewer(s). You should upload this letter as a separate file labeled 'Response to Reviewers'.
- A marked-up copy of your manuscript that highlights changes made to the original version. You should upload this as a separate file labeled 'Revised Manuscript with Track Changes'.
- An unmarked version of your revised paper without tracked changes. You should upload this as a separate file labeled 'Manuscript'.

If you would like to make changes to your financial disclosure, please include your updated statement in your cover letter. Guidelines for resubmitting your figure files are available below the reviewer comments at the end of this letter.

If applicable, we recommend that you deposit your laboratory protocols in protocols.io to enhance the reproducibility of your results. Protocols.io assigns your protocol its own identifier (DOI) so that it can be cited independently in the future. For instructions see: https://journals.plos.org/plosone/s/submission-guidelines#loc-laboratory-protocols. Additionally, PLOS ONE offers an option for publishing peer-reviewed Lab Protocol articles, which describe protocols hosted on protocols.io. Read more information on sharing protocols at https://plos.org/protocols?utm_medium=editorial-email&utm_source=authorletters&utm_campaign=protocols.

As the corresponding author, your ORCID iD is verified in the submission system and will appear in the published article. PLOS supports the use of ORCID, and we encourage all coauthors to register for an ORCID iD and use it as well. Please encourage your coauthors to verify their ORCID iD within the submission system before final acceptance, as unverified ORCID iDs will not appear in the published article. *Only*  the individual author can complete the verification step; PLOS staff *cannot*  verify ORCID iDs on behalf of authors.

We look forward to receiving your revised manuscript.

Kind regards,

Raed Abduljabbar Haleem, Ph.D

Academic Editor

PLOS One

Journal Requirements:

When submitting your revision, we need you to address these additional requirements.

1. Please ensure that your manuscript meets PLOS ONE's style requirements, including those for file naming. The PLOS ONE style templates can be found at

https://journals.plos.org/plosone/s/file?id=wjVg/PLOSOne_formatting_sample_main_body.pdf and https://journals.plos.org/plosone/s/file?id=ba62/PLOSOne_formatting_sample_title_authors_affiliations.pdf

2. In your Methods section, please provide additional information regarding the permits you obtained for the work. Please ensure you have included the full name of the authority that approved the field site access and, if no permits were required, a brief statement explaining why.

3. Please provide a complete Data Availability Statement in the submission form, ensuring you include all necessary access information or a reason for why you are unable to make your data freely accessible. If your research concerns only data provided within your submission, please write "All data are in the manuscript and/or supporting information files" as your Data Availability Statement.

4. Thank you for stating the following financial disclosure:

Doctor Professor Service Group" Special Project of Kaili University (2024, No. BS20240214); the Integrated Research Team for Resource Utilization and Fauna & Flora Conservation of Kaili University (2025, No. YTH-TD20255I); the Natural Science Research Youth Project of the Department of Education of Guizhou Province (2024, No. Qian Jiaoji [2024]230); and Youth Science and Technology Innovation Fund(XXNK202543)

Please state what role the funders took in the study. If the funders had no role, please state: "The funders had no role in study design, data collection and analysis, decision to publish, or preparation of the manuscript."

If this statement is not correct you must amend it as needed.

Please include this amended Role of Funder statement in your cover letter; we will change the online submission form on your behalf.

5. Please update your submission to use the PLOS LaTeX template. The template and more information on our requirements for LaTeX submissions can be found at http://journals.plos.org/plosone/s/latex.

6. Please ensure that you refer to Figure 1 in your text as, if accepted, production will need this reference to link the reader to the figure.

7. Please include a caption for figure 1.

8. If the reviewer comments include a recommendation to cite specific previously published works, please review and evaluate these publications to determine whether they are relevant and should be cited. There is no requirement to cite these works unless the editor has indicated otherwise.

[Note: HTML markup is below. Please do not edit.]

Reviewers' comments:

Reviewer's Responses to Questions

**Comments to the Author**

1. Is the manuscript technically sound, and do the data support the conclusions?

The manuscript must describe a technically sound piece of scientific research with data that supports the conclusions. Experiments must have been conducted rigorously, with appropriate controls, replication, and sample sizes. The conclusions must be drawn appropriately based on the data presented.

Reviewer #1: Yes

Reviewer #2: Yes

Reviewer #3: Partly

**********

2. Has the statistical analysis been performed appropriately and rigorously?

Reviewer #1: N/A

Reviewer #2: N/A

Reviewer #3: No

**********

3. Have the authors made all data underlying the findings in their manuscript fully available?

The PLOS Data policy requires authors to make all data underlying the findings described in their manuscript fully available without restriction, with rare exception (please refer to the Data Availability Statement in the manuscript PDF file). The data should be provided as part of the manuscript or its supporting information, or deposited to a public repository. For example, in addition to summary statistics, the data points behind means, medians and variance measures should be available. If there are restrictions on publicly sharing data—e.g. participant privacy or use of data from a third party—those must be specified.

Reviewer #1: Yes

Reviewer #2: Yes

Reviewer #3: No

**********

4. Is the manuscript presented in an intelligible fashion and written in standard English?

PLOS ONE does not copyedit accepted manuscripts, so the language in submitted articles must be clear, correct, and unambiguous. Any typographical or grammatical errors should be corrected at revision, so please note any specific errors here.

Reviewer #1: Yes

Reviewer #2: Yes

Reviewer #3: No

**********

5. Review Comments to the Author

Please use the space provided to explain your answers to the questions above. You may also include additional comments for the author, including concerns about dual publication, research ethics, or publication ethics. (Please upload your review as an attachment if it exceeds 20,000 characters)

Reviewer #1: The manuscript titled "First Report of Corynespora cassiicola Causing Leaf Spot on Begonia fimbristipula in China" presents valuable findings, but several critical issues must be addressed before it can be considered for publication. My comments are structured as follows:

1. General Summary & Significance

This study investigates a severe leaf spot disease affecting Begonia fimbristipula, an important medicinal plant in southern China, resulting in significant yield losses. The core hypothesis is that an uncharacterized pathogen is responsible for this disease. The methodology employed field surveys to document disease incidence and symptoms, followed by isolation of the causative agent. Identification was achieved through a polyphasic approach combining detailed morphological characterization with multilocus phylogenetic analysis based on ITS, TEF1α, and RPB2 sequences. The pathogenicity of the identified organism was rigorously confirmed by fulfilling Koch's postulates through artificial inoculation and re-isolation. The findings indicate that the outbreaks observed in August 2024 resulted in over 60% disease incidence and >20% loss of fresh leaf yield. The pathogen was conclusively identified as Corynespora cassiicola, based on its distinctive morphological features and strong phylogenetic clustering with known reference strains. Artificial inoculation successfully reproduced the characteristic symptoms, and the pathogen was successfully re-isolated, thereby confirming its causal role.

This study holds significant value as it constitutes the first documented report of Corynespora cassiicola causing leaf spot on Begonia fimbristipula in China. This finding is original as it directly extends the known host range of this economically important and cosmopolitan fungal pathogen, providing valuable data for plant pathology and host-pathogen interaction studies. The scientific rigor of the study is commendable, particularly in its use of a robust polyphasic identification approach and the strict adherence to Koch's postulates for pathogenicity confirmation. The inclusion of multiple molecular markers (ITS, TEF1α, RPB2) strengthens the reliability of the molecular identification. The work provides a crucial foundational understanding for diagnosing and developing integrated management strategies for this newly emerging disease affecting a valuable medicinal plant.

2. Structure & Presentation

The title, "First Report of Corynespora cassiicola Causing Leaf Spot on Begonia fimbristipula in China," is adequate, appropriate, and accurately reflects the study's content. The abstract effectively covers all essential components: background, methods, results, and conclusion. It is comprehensive enough to stand alone for abstracting services.

The manuscript is generally well-written and the scientific information is communicated clearly. The language is understandable, although there are several instances of awkward phrasing, minor grammatical errors, and slightly repetitive sentences that detract from the overall polish expected for a high-impact journal.

Specific examples of awkward phrasing/grammar:

- In the Abstract's background, the sentence "Leaf spot disease has recently caused severe yield losses to B. fimbristipula in Guizhou and Hunan Provinces, but the causal pathogen remains unknown" could be rephrased for better flow, e.g., "...a significant leaf spot disease has recently emerged on B. fimbristipula in Guizhou and Hunan Provinces, causing severe yield losses, but its causal pathogen remains unidentified."

- In the Methodology section for PCR Amplification and Sequencing, the description of the PCR program for ITS contains a repetitive element: "...72°C extension for 1 min; and 72°C final extension for 10 min." This should be streamlined.

- The caption for Figure S1 (Fig S1 A1, A2, A3...) is grammatically fragmented and needs to be rewritten as complete, descriptive sentences.

A thorough revision to improve grammatical precision and eliminate minor redundancies would enhance conciseness. For example, the detailed descriptions of DNA extraction and PCR sequencing protocols are repeated almost verbatim in the abstract and the main Methods section, and are implicitly present in the Results. This should be streamlined, with full details appearing only once in the Methods section.

Visuals: The intent of the visuals (Figure S1 showing symptoms, Koch's postulates, colony, and conidia; Figure S2 showing a phylogenetic tree) is excellent and they are essential to support the findings. However, their presentation needs significant improvement:

Clarity and Quality: The embedded images (Figures S1 and S2) are low-resolution, making it difficult to discern important details (e.g., fungal structures, precise symptoms, and bootstrap values on the phylogenetic tree). High-resolution images are imperative for publication.

-The caption for Figure S1 is not understandable and requires complete rewriting to accurately and clearly describe each panel.

-A major inconsistency exists with Figure S2: the main text states phylogenetic analysis was performed using the "neighbor-joining (NJ) method," while the figure caption states "using Maximam Likelihood method." This is a critical contradiction that must be resolved. Ensure all details, particularly morphological features in S1 and bootstrap values/branching patterns in S2, are clearly legible. Completely rewrite the caption for Figure S1. Each panel (A1-A3, B1-B4, C, D) must be described clearly and concisely using full, grammatically correct sentences. Ensure the caption for Figure S2 is also clear and accurate, matching the text.

3. Section-Specific Comments

Introduction: The introduction is comprehensive and well-structured. It effectively establishes the economic and medicinal importance of B. fimbristipula, introduces the emerging disease problem, and adequately reviews existing literature on C. cassiicola's host range. The authors clearly delineate the knowledge gap that their study addresses, providing a strong rationale for the research.

Methodology: The methodologies employed are scientifically sound, valid, and based on established plant pathology techniques for pathogen isolation, identification, and pathogenicity testing. Refer to https://doi.org/10.3390/v11070588 as justification for using specific Phylogenetic Analysis as a more robust method for examining the evolutionary distance between C. cassiicola isolates. Ensure that the method stated in the main text (e.g., "neighbor-joining (NJ) method") perfectly matches the method specified in the Figure S2 caption (currently " Maximum Likelihood method). Provide a brief justification for the chosen method.

The DNA extraction kit, PCR primers, concentrations, and programs are outlined. GenBank accession numbers are provided, which is excellent for data transparency. However, the critical contradiction regarding the phylogenetic method between the text and Figure S2 caption must be resolved to ensure reproducibility and credibility. Clarification is also needed on whether all 10 isolates underwent molecular identification or if ZBTK-1 was exclusively characterized, with justification if the latter. Explicitly state whether molecular identification was performed on all 10 representative isolates (ZBTK1-ZBTK10) or solely on isolate ZBTK-1. If only one isolate was fully sequenced, provide a clear scientific justification for this approach (e.g., genetic redundancy, resource limitations, or ZBTK-1 being representative after preliminary screening of all 10).

For the control, specifying "sterile PDA plugs without mycelia would add clarity.

Results: The results are credible and logically presented, supported by the analytical methods described. Descriptions of symptoms are clear. The reported incidence (60%) and yield loss (>20%) are impactful, though a brief explanation of how these figures were estimated (even if observational) would enhance their validity.

In the "Field Disease Symptoms and Incidence" subsection, briefly describe the methodology used to estimate the "over 20% fresh leaf yield loss." Even if qualitative, outlining the observational criteria would add credibility (e.g., visual assessment scale, sampled areas, etc.).

Consider Quantitative Pathogenicity Data: If quantitative data on disease severity (e.g., lesion diameter measurements, disease index scores) were collected during the pathogenicity assays, incorporate them into the Results section. It would significantly strengthen the evidence for pathogenicity. If not collected, state that qualitative visual assessments were deemed sufficient.

Discussion: The discussion is logical, comprehensive, and effectively connects the findings to their broader implications. It goes beyond merely restating the results by contextualizing them within existing literature, emphasizing the expanded host range of C. cassiicola, and discussing its potential impact on B. fimbristipula cultivation and regional agriculture. The proposed integrated management strategies (cultural, chemical, resistance breeding) are relevant and practical. However, I suggest expanding the study's limitations to include the complex biochemical interactions—such as how the pathogen might manipulate host cell signaling or phytohormone pathways to overcome plant immunity (refer to https://doi.org/10.15414/jmbfs.3422)—that remain to be elucidated, beyond identifying the causal agent.

Conclusion: The conclusion is well-written and succinctly summarizes the key takeaway.

Reviewer #2: Comments

First Report of Corynespora cassiicola Causing Leaf Spot on Begonia fimbristipula in China

� The sentence provides useful context, but it is slightly descriptive for an abstract. It could be more concise while retaining key relevance.

� “severe yield losses” is vague and could be strengthened with brief quantitative indication.

� Brief mention of scale or scope would improve the methodological clarity.

� This is a strong and informative result statement, effectively conveying the scale and impact of the disease

� It could be made more concise by avoiding repetition of procedural details.

� The statement is slightly overstated, as management aspects are not directly addressed within the abstract.

� slightly lengthy. It could be streamlined for better readability while retaining key information.

� Including examples of diseases or brief statistics would strengthen the argument.

� This provides clear context and significance; however, quantitative data on yield loss would improve precision.

� listing multiple hosts may slightly reduce conciseness.

� The claim regarding disease control is somewhat broad without direct supporting evidence.

� The survey duration and frequency are not described.

� The parameters are relevant, but methods for measuring incidence and yield loss are not explained.

� Justification for sterilization duration is not provided

� “±0°C” appears unrealistic and may indicate a reporting error.

� It lacks detail on how single spores were isolated and confirmed.

� criteria for choosing representative isolates are not specified.

� The measurement approach is appropriate, but increasing sample size could improve statistical reliability.

� Reliance solely on comparison may introduce subjectivity without clear criteria.

� justification for selecting only one isolate is lacking.

� kit-based methods may limit methodological transparency.

� Thermal cycling conditions are clearly described, though minor redundancy could be reduced for conciseness

� This is a standard validation step, but lacks details such as voltage and run time.

� Providing accession numbers enhances transparency and data availability.

� More robust approaches (e.g., ML or Bayesian) could strengthen analysis.

� no justification for model selection is provided.

� plant selection criteria is not described.

� Wounding may artificially enhance infection and should be justified or discussed.

� Inclusion of a control is appropriate and strengthens experimental validity

� statistical analysis of results is not mentioned.

� Criteria for symptom assessment are not specified.

� The approach is methodologically sound and essential for confirming causality.

� The use of a single representative isolate simplifies analysis but may not capture genetic variability among isolates.

� Reliance on commercial kits limits methodological transparency.

� Minor redundancy could be reduced.

� This is a routine step, but lacks details such as electrophoresis conditions.

� No justification for concatenation strategy is provided.

� It lacks justification for plant age selection.

� The absence of wounding increases realism, though infection efficiency may vary.

� statistical analysis is not mentioned.

� Observation frequency is appropriate, but scoring criteria are not defined.

� Quantitative data effectively demonstrate disease severity and impact.

� Symptom description is clear and detailed, aiding disease identification.

� The conclusion is appropriate but relies on descriptive comparison.

� This validates the experimental setup and strengthens conclusions.

� The statement is valid but somewhat general.

� This is a reasonable inference, though not experimentally tested.

� The statement is justified but slightly overstated without applied data.

� Suggestions are relevant but remain hypothetical.

� The authors appropriately acknowledge key limitations.

� The statement is relevant but slightly broad.

Reviewer #3: This manuscript reports the identification of Corynespora cassiicola as the causal agent of leaf spot disease on Begonia fimbristipula, based on field observations, morphological characterization, multilocus phylogenetic analysis (ITS, TEF1α, and RPB2), and pathogenicity assays fulfilling Koch’s postulates. The study addresses a relevant phytopathological issue, particularly considering the economic and medicinal importance of B. fimbristipula in southern China, and contributes to documenting disease occurrence in this host.

Overall, the experimental framework adopted by the authors follows a generally accepted approach for pathogen identification, combining classical mycological methods with molecular phylogenetics and pathogenicity testing. The successful fulfillment of Koch’s postulates and the multilocus identification provide support for the conclusion that C. cassiicola is associated with the observed disease symptoms. However, despite this technically valid foundation, the manuscript in its current form presents several limitations that need to be addressed before it can be considered for publication.

A central issue is that the study remains largely descriptive, with limited quantitative analysis to support the conclusions. In particular, the pathogenicity assays are described qualitatively, with visual confirmation of symptom development, but without quantitative measurement of disease severity (e.g., lesion size, lesion number per unit area, or severity indices) and without statistical analysis. The inclusion of quantitative disease assessments and appropriate statistical comparisons would substantially strengthen the robustness and reproducibility of the findings.

Another important limitation concerns the phylogenetic analysis. Although multiple isolates were obtained from infected tissues, only a single representative isolate appears to have been used for multilocus sequencing and phylogenetic reconstruction. This restricts the ability to assess intra-population variability and limits the generalization of the identification. The authors should clarify the rationale for selecting a single isolate and, if possible, include additional isolates in the phylogenetic analysis or explicitly acknowledge this limitation in the discussion.

The manuscript also presents structural issues that affect clarity. Notably, sections of the Methods describing molecular identification appear to be duplicated, which disrupts the logical flow of the manuscript and should be corrected. In general, the organization of the Methods section would benefit from careful revision to improve coherence and avoid redundancy.

Regarding statistical analysis, the manuscript includes only minimal quantitative treatment of the data. While descriptive statistics are provided for morphological measurements, there is no statistical evaluation of pathogenicity results or comparison among isolates. As such, the statistical analysis cannot be considered rigorous in its current form. The authors should either incorporate appropriate statistical analyses where relevant or clearly justify the descriptive nature of the study.

Data availability is another aspect that requires clarification. While sequence data appear to be deposited in public databases, it is not fully clear whether all data underlying the findings (e.g., raw measurements, experimental observations, or additional datasets) are available, as required by PLOS ONE data policies. The Data Availability statement should be revised to explicitly indicate where all supporting data can be accessed.

The interpretation of the results, particularly in the Discussion and Conclusion sections, should also be moderated. Some statements extend beyond the data presented, especially those related to broader implications for disease management, crop protection, and resistance breeding. While these perspectives are relevant, they are not directly supported by experimental evidence in this study and should be presented more cautiously as potential implications rather than demonstrated outcomes.

In addition, the manuscript requires substantial improvement in English language quality. Numerous grammatical errors, awkward sentence constructions, and redundancies affect readability and clarity. Careful language editing by a fluent English speaker or professional editing service is strongly recommended to meet the journal’s standards.

Despite these limitations, the study has several strengths. It addresses an underreported host-pathogen interaction, applies a combination of classical and molecular identification approaches, and provides baseline information that may be useful for future epidemiological and phytopathological studies. With improved data presentation, clearer methodological description, and more cautious interpretation of results, the manuscript could represent a useful contribution.

In summary, the study is technically sound in its core approach but only partially supports its conclusions in its current form due to limitations in quantitative analysis, statistical rigor, and presentation. I therefore recommend major revision, with particular emphasis on strengthening data analysis, improving methodological clarity, and refining the interpretation and language of the manuscript.

**********

6. PLOS authors have the option to publish the peer review history of their article (what does this mean?). If published, this will include your full peer review and any attached files.

If you choose “no”, your identity will remain anonymous but your review may still be made public.

**Do you want your identity to be public for this peer review?**  For information about this choice, including consent withdrawal, please see our Privacy Policy.

Reviewer #1: No

Reviewer #2: No

Reviewer #3: No

**********

[NOTE: If reviewer comments were submitted as an attachment file, they will be attached to this email and accessible via the submission site. Please log into your account, locate the manuscript record, and check for the action link "View Attachments". If this link does not appear, there are no attachment files.]

To ensure your figures meet our technical requirements, please review our figure guidelines: https://journals.plos.org/plosone/s/figures

You may also use PLOS’s free figure tool, NAAS, to help you prepare publication quality figures: https://journals.plos.org/plosone/s/figures#loc-tools-for-figure-preparation.

NAAS will assess whether your figures meet our technical requirements by comparing each figure against our figure specifications.

Attachment Submitted filename: Reviewed File.docx

# Author comment: Author response to Decision Letter 1

**DOI:** 10.1371/journal.pone.0346996.r002

20 May 2026

Responses to Reviewer #1

Comment 1: Language and grammar issues.

Response: The entire manuscript has been edited for clarity. The PCR program description has been streamlined, and the duplicate section removed. Figure captions rewritten.

Comment 2: Low-resolution figures; contradiction in phylogenetic method.

Response: We have replaced the figures with the best resolution attainable from our original image files. The method is now consistently described as “neighbor-joining (NJ) with 1000 bootstrap replicates” in both text and figure caption.

Comment 3: Yield loss estimation method not described.

Response: Added: “Yield loss was estimated by field observation comparing symptomatic vs. asymptomatic plants in representative plots.”

Comment 4: Clarify whether all 10 isolates were sequenced.

Response: Clarified in Methods: “Only ZBTK-1 was used for multilocus sequencing because ITS sequences of all 10 isolates were 100% identical (data not shown).” Also noted as a limitation in Discussion.

Comment 5 (suggested citation): Added as requested.

Responses to Reviewer #2

Reviewer #2 raised many short points. We address the main categories:

Missing methodological details (survey duration, incidence calculation, sterilization justification, isolate selection, plant age): Added to Methods using existing records. No new experiments.

Statistical analysis: We state that pathogenicity was assessed qualitatively, which is standard for first reports. No statistical tests were performed. A limitation statement added.

Redundancy: Duplicate section removed.

“±0°C” : Corrected to “25 ± 1°C”.

Responses to Reviewer #3

Comment 1 (quantitative pathogenicity data and statistics):

We thank the reviewer for the suggestion. However, in the context of a First Report, the standard requirement for pathogenicity confirmation is qualitative fulfillment of Koch’s postulates – i.e., symptom reproduction and re-isolation – without mandatory quantitative metrics or statistical analysis. This is consistent with countless First Reports published in Plant Disease, Journal of Plant Pathology, and other journals, including previous reports of C. cassiicola on new hosts (e.g., references [2–5] in our manuscript). Therefore, in keeping with standard practice for First Reports, we did not perform quantitative lesion measurements. We have, however, explicitly acknowledged this as a limitation in the Discussion and clarified in Methods that assessment was qualitative. We hope the reviewer will accept this as sufficient for a first report.

Comment 2 (single isolate for multilocus sequencing):

We acknowledge the limitation. We have clarified that ITS sequences of all 10 isolates were identical, and that only one isolate was fully sequenced due to resource constraints. A limitation statement added in Discussion.

Comment 3 (duplicated Methods):

Deleted.

Comment 4 (statistical analysis minimal):

As noted above, the study is descriptive; no inferential statistics were performed. We have stated this explicitly in Methods and Discussion.

Comment 5 (data availability):

Data Availability Statement updated. All sequence data are in GenBank. Morphological data (conidial dimensions) are reported in Results. No other raw data were collected.

Comment 6 (overstatements in Discussion/Conclusion):

We have significantly moderated the language. Management suggestions are now presented as future research directions, not as demonstrated outcomes. Conclusion revised to remove claims about integrated management.

Comment 7 (English language):

Manuscript has been carefully edited.

Attachment Submitted filename: Response to Reviewers.docx

# Editor report: Decision Letter 1

**DOI:** 10.1371/journal.pone.0346996.r003

**Authors:** Raed Haleem

24 May 2026

First Report of Corynespora cassiicola Causing Leaf Spot on Begonia fimbristipula in China

PONE-D-26-14713R1

Dear Dr. Lu,

We’re pleased to inform you that your manuscript has been judged scientifically suitable for publication and will be formally accepted for publication once it meets all outstanding technical requirements.

Within one week, you’ll receive an e-mail detailing the required amendments. When these have been addressed, you’ll receive a formal acceptance letter and your manuscript will be scheduled for publication.

An invoice will be generated when your article is formally accepted. Please note, if your institution has a publishing partnership with PLOS and your article meets the relevant criteria, all or part of your publication costs will be covered. Please make sure your user information is up-to-date by logging into Editorial Manager at Editorial Manager® and clicking the ‘Update My Information' link at the top of the page. For questions related to billing, please contact billing support.

If your institution or institutions have a press office, please notify them about your upcoming paper to help maximize its impact. If they’ll be preparing press materials, please inform our press team as soon as possible -- no later than 48 hours after receiving the formal acceptance. Your manuscript will remain under strict press embargo until 2 pm Eastern Time on the date of publication. For more information, please contact onepress@plos.org.

Kind regards,

Raed Abduljabbar Haleem, Ph.D

Academic Editor

PLOS One

Reviewers' comments:

# Editor report: Acceptance letter

**DOI:** 10.1371/journal.pone.0346996.r004

**Authors:** Raed Haleem

PONE-D-26-14713R1

PLOS One

Dear Dr. Lu,

I'm pleased to inform you that your manuscript has been deemed suitable for publication in PLOS One. Congratulations! Your manuscript is now being handed over to our production team.

At this stage, our production department will prepare your paper for publication. This includes ensuring the following:

* All references, tables, and figures are properly cited

* All relevant supporting information is included in the manuscript submission,

* There are no issues that prevent the paper from being properly typeset

You will receive further instructions from the production team, including instructions on how to review your proof when it is ready. Please keep in mind that we are working through a large volume of accepted articles, so please give us a few days to review your paper and let you know the next and final steps.

Lastly, if your institution or institutions have a press office, please let them know about your upcoming paper now to help maximize its impact. If they'll be preparing press materials, please inform our press team within the next 48 hours. Your manuscript will remain under strict press embargo until 2 pm Eastern Time on the date of publication. For more information, please contact onepress@plos.org.

You will receive an invoice from PLOS for your publication fee after your manuscript has reached the completed accept phase. If you receive an email requesting payment before acceptance or for any other service, this may be a phishing scheme. Learn how to identify phishing emails and protect your accounts at https://explore.plos.org/phishing.

If we can help with anything else, please email us at customercare@plos.org.

Thank you for submitting your work to PLOS ONE and supporting open access.

Kind regards,

PLOS ONE Editorial Office Staff

on behalf of

Dr. Raed Abduljabbar Haleem

Academic Editor

PLOS One
